# Recovery of DNA of *Giardia intestinalis* cysts from surface water concentrates measured with PCR and real time PCR

**DOI:** 10.1051/parasite/2011184341

**Published:** 2011-11-15

**Authors:** M. Adamska, A. Leońska-Duniec, A. Maciejewska, M. Sawczuk, B. Skotarczak

**Affiliations:** 1 Department of Genetics, University of Szczecin ul. Felczaka 3c 71-412 Szczecin Poland

**Keywords:** DNA extraction, *Giardia intestinalis*, PCR, real time PCR, détection d’ADN, *Giardia intestinalis*, PCR, PCR en temps réel

## Abstract

The most important restriction for the detection in water samples is the low concentration of *Giardia intestinalis* cysts, additional difficulty is the presence of PCR inhibitors. We have carried out trials in order to assess the sensitivity of semi-nested PCR and TaqMan real time PCR on the basis of DNA extracted from *G. intestinalis* cysts coming from spiked environmental and distilled water samples, filtrated with the use of Filta-Max^®^ equipment (1623 Method). Removal of inhibitors was carried out with addition of BSA in different concentrations. During the filtration and concentration of water samples, losses of cysts have been recorded. Moreover, addition of BSA to the PCR and real time PCR mix increases the sensitivity of reaction. The optimal concentration of BSA for semi-nested PCR was 15 and 20 ng/μl, whereas for real time PCR 5 ng/μl.

## Abstract (fr)

La faible concentration des kystes de *Giardia intestinalis* dans les prélèvements d’eau de surface, ainsi que la présence d’inhibiteurs de la PCR constituent un handicap pour la détection du parasite. Nous avons effectué une série d’essais afin de tester la sensibilité de la PCR semi-nichée et la TaqMan PCR en temps réel dans la détection de l’ADN extrait de kystes de *G. intestinalis*. Ces kystes proviennent d’échantillons d’eau distillée et d’eau d’environnement, filtrés avec le Filta-Max^®^ (1623 Method). Les inhibiteurs ont été éliminés par la BSA utilisée à différentes concentrations. Des pertes de kystes ont été constatées lors des opérations de filtration et de concentration. L’addition de BSA augmente la sensibilité de la réaction. La concentration optimale de BSA était de 15 et 20 ng/μl pour la PCR semi-nichée et de 5 ng/μl pour la PCR en temps réel.

*Giardia intestinalis* is an intestinal unicellular parasite causing diarrhea in humans worldwide. The transmission occurs mainly through water contaminated with *Giardia* cysts (Castro- Hermida et al., 2008; Haque *et al.*, 2007). Giardiasis is generally self-limiting and asymptomatic in healthy individuals; however, the infection may be a serious health risk for immunocompromised individuals and for children. Although giardiasis is a serious public health problem, the diagnosis of this disease is not routinely approved (Thompson, 2004).

In order to improve the monitoring of *Giardia* cysts in water, the United States Environmental Protection Agency introduced the 1623 Method (EPA, 2005). This method, used to determine the presence and concentration of cysts in water, is based on microscopic detection; however, more sensitive methods are required for the detection of the very low concentrations of the parasite in water and the identification of the species (Guy *et al.*, 2003). Molecular methods give such possibilities, but substances contained in water samples can be potential inhibitors in tests based on DNA.

In an anterior study, we evaluated the efficiencies of different DNA extraction methods (Adamska *et al.*, 2010). In the present study, we have carried out trials to assess the sensitivity of semi-nested PCR and TaqMan real time PCR on the basis of DNA extracted from *G. intestinalis* cysts providing from spiked environmental and distilled water samples. Moreover, the addition of BSA in different concentrations was applied to remove inhibitors.

## Materials and Methods

### DNA Isolation From Spiked Water Samples

Samples containing 8 × 105 cysts of *G. intestinalis* (Bulk Stock Live, BTF Biomérieux, Australia) in PBS were used for DNA extraction. Spiked samples were prepared by adding solutions containing about 8 × 105 cysts in 10 l of distilled water, and in 10 l of environmental water (Glebokie Lake). They were subjected to this procedure with Manual Filta-Max® Wash Station (IDEXX, USA), according to the manufacturer’s instructions. 300 μl of eluate were collected for the extraction as described previously (Adamska *et al.*, 2010).

### Semi-Nested PCR and TaqMan Real Time PCR Amplification

A region of the β-giardin gene was amplified by semi-nested PCR protocol described by Caccio et al. (2002). PCR mixture and thermal-time profiles were described previously by Adamska *et al*. (2010) and Castro-Hermida *et al.* (2008), respectively. Additionaly, bovine serum albumin (BSA) was added to PCR mix in concentrations: 0, 5, 10, 15, 20, 50, 100, 200 and 400 ng/μl. Negative control mixtures contained sterile distilled water in place of DNA. PCR products were visualized by 1.5 % agarose gel electrophoresis. All analyses were carried out in four replicates.

A region of the small subunit rRNA gene of *G. intestinalis* was used as a target sequence for TaqMan real time PCR (Haque *et al.*, 2007). PCR mixture and thermal-time profiles were the same as described by Adamska et al. (2010) and Haque *et al.* (2007), respectively. Additionaly, BSA was added to PCR mix in concentrations: 0, 5, 10, 15, 20, 50, 100, 200 and 400 ng/μl. Appropriate negative controls were included in each PCR run. All analysis were carried out in four replicates.

## Results and Discussion

The intensity of signal obtained with semi-nested PCR was similar for isolates received from filtration of spiked distilled and lake water, whereas intensity was higher when isolates were directly received from *G. intestinalis* cysts in PBS. The seminested PCR results have been confirmed by TaqMan real time PCR in form of adequate CT values, which were similar in isolates from distilled (22.21 ± 0.34) and lake (24.38 ± 0.49) water, whereas CT values for isolates from *G. intestinalis* cysts in PBS were lower (16.14 ± 0.23).

**Fig. 1.**
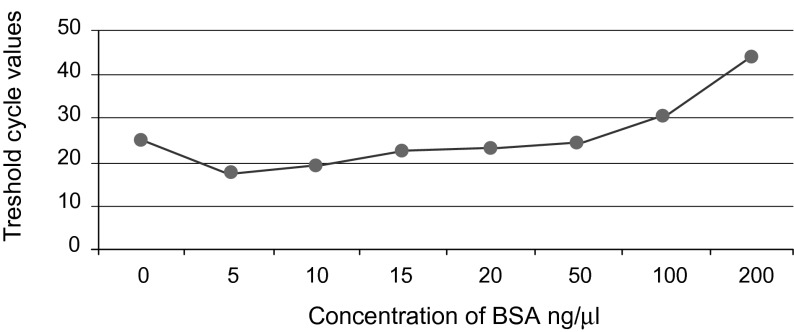
Correlation between CT values and BSA concentrations for TaqMan real time PCR.

Addition of BSA to PCR mix for samples isolated from spiked lake water caused an increase in reaction sensitivity. For semi-nested PCR, we obtained very strong intensity of DNA bands for 15 and 20 ng/μl, strong for 5, 10 and 50 ng/μl, medium for 0 ng/μl and weak for 100 ng/μl. There were no PCR signals for 200, 300 and 400 ng/μl. CT values for real time PCR with addition of BSA are shown in Fig. 1.

Earlier studies have shown various degrees of recovery efficiency for *Giardia* and noted a decrease in the recovery level of cysts after filtration (Wohlsen *et al.*, 2004). In our study, losses of *Giardia* cysts were also recorded during the filtration of spiked distilled and lake water samples. The intensity of signal obtained with semi-nested PCR method was similar for isolates received from filtration of spiked distilled and lake water, whereas in case isolates received directly from *G. intestinalis* cysts in PBS, the intensity was higher. The semi-nested PCR results have been confirmed by real time PCR results in form of adequate CT values.

The environmental samples are rich in PCR inhibitors which could be co-extracted with DNA and which could interfere with the PCR amplification (Jiang *et al.*, 2005; Schriewer *et al.*, 2011). According to the 1623 Method, the next step after concentration of *Giardia* cysts is the immunomagnetic separation (IMS) that should eliminate the inhibitors (USEPA 2005). However, this method becomes impractical for organisms that have no IMS procedure, and is also expensive (Jiang *et al.*, 2005). Many chemicals are used to deactivate PCR inhibitors, *e.g.* bovine serum albumin (BSA) that is one of the most efficient facilitators. BSA has been widely used to reduce inhibitory effects in conventional PCR assays of protozoan, however, studies reporting optimization of BSA concentrations for real time PCR are rare (Jiang *et al.*, 2005; Schriewer *et al.*, 2011).

Many different BSA concentrations were used in earlier studies (from 20 to 400 ng/μl) (Jiang *et al.*, 2005; Schriewer *et al.*, 2011). Studies of Guy *et al.* (2003) revealed that addition of BSA in concentration 20 ng/μl to the real time PCR mixture removed the inhibitory effect. In our study, addition of BSA to PCR mix for samples isolated from spiked lake water caused an increase in reaction sensitivity, but only at low concentrations. For semi-nested PCR, the optimal concentration of BSA was 15 and 20 ng/μl, whereas for TaqMan real time PCR 5 ng/μl. Our studies revealed that high concentration of BSA (100 ng/μl and above) has an inhibiting effect on the PCR. Schriewer *et al.* (2011) obtained similar results. However, according to Jiang *et al.* (2005), the effect of PCR inhibitors was significantly reduced with 400 ng/μl. The optimum range of applied BSA may vary depending on the amount of inhibitors and the matrix composition (Schriewer *et al.*, 2011), so the origin of environmental sample and type of detected organism may affect optimum BSA concentration.
